# Improvement of Inter-Professional Collaborative Work Abilities in Mexican Medical and Nursing Students: A Longitudinal Study

**DOI:** 10.3389/fpsyg.2019.00005

**Published:** 2019-01-15

**Authors:** Guillermo J. Tuirán-Gutiérrez, Montserrat San-Martín, Roberto Delgado-Bolton, Blanca Bartolomé, Luis Vivanco

**Affiliations:** ^1^School of Healthcare Sciences, Autonomous University of Coahuila, Piedras Negras, Mexico; ^2^Faculty of Social Sciences of Melilla, University of Granada, Melilla, Spain; ^3^Center for Biomedical Research of La Rioja (CIBIR), Logroño, Spain; ^4^National Centre of Documentation on Bioethics, Logroño, Spain

**Keywords:** professionalism, empathy, lifelong learning, inter-professional collaborative work, medical students, nursing students, professional roles

## Abstract

**Background:** Inter-professional and interpersonal relationships in collaborative work environments can prove to be critical elements in healthcare practice. When implementers fail to understand the importance of a collaborative perspective, this can lead to communication problems which ultimately harm the users.

**Objectives:** To improve the inter-professional collaborative work skills of Mexican students in their first year of medical and nursing degrees through the use of a training program geared toward development of interpersonal skills and interdisciplinary work.

**Methods:** The sample was composed of 162 students (62 males and 99 females) from the School of Healthcare Sciences of the Autonomous University of Coahuila, Mexico. The main measures used were the Jefferson Scale of Empathy (JSE); the Jefferson Scale of Attitudes toward Inter-Professional Collaborative Work between Medical and Nursing Professionals (JSAPNC); and the Jefferson Scale of Lifelong Learning (JeffSPLL). The entire sample was divided into two groups (experimental and control groups). Both groups attended an extra-curricular program using a coaching methodology. In the first case the topic focused on attitudes toward inter-professional collaborative work. In the second case, the program focused on addiction. Both programs ran for 4 months. Psychometric instruments were applied at the beginning and at the end of both programs. After analyzing the reliability of the instruments, an ANOVA test was performed.

**Results:** The control group of medical students showed a deterioration in the development of collaborative work skills (*p* < 0.01), whereas in the experimental group this deterioration was not present. In the experimental group of nursing students, a significant increase in the development of collaborative work skills (*p* < 0.05) was observed. The differences were clearly due to the professional area of study (*p* < 0.001).

**Conclusion:** There are differences in collaborative work skill development among different professional areas. These differences can be reduced through the implementation of a program aimed at developing collaborative work and interpersonal skills in the early stages of training.

## Introduction

In physicians and healthcare professionals, professionalism refers to the set of skills and values that characterizes the essence of humanism in professional work (Vivanco and Delgado-Bolton, 2015). This concept comprises an articulated body of professional traits and skills that constitute the essence of their professional work regardless of their geographical, social, or cultural settings. For assessment purposes, a definition from the medical research field suggests that medical professionalism is achieved by mastering three essential areas: clinical skills, communication skills, and an appropriate understanding of the ethical and legal framework of professional behavior (Stern, 2006). Communication skills are not limited to patients and their families, but also relate to other healthcare professionals who are part of the inter-professional team which works at the healthcare institutions. According to the World Health Organization (2010), this inter-professional collaboration, also called teamwork, is described as the ability of “multiple healthcare workers from different professional backgrounds to provide comprehensive services by working with patients, their families, their professional careers, and the community, in order to deliver the highest quality of healthcare across settings.” Benefits of this teamwork have been described in recent publications in healthcare institutions (Pedrazza et al., 2017). Some of those benefits are associated with the improvement of certain professional competences that are important in interdisciplinary teams′ performance, such as communication, innovation, and creativity skills or with decision-making abilities (Ceschi et al., 2014). In fact, according to Veloski and Hojat (2006), teamwork, together with empathy and lifelong learning, are the three essential components of professionalism in medical and healthcare sciences.

Despite important social, political, and economical differences among Latin American countries, there are no big differences in terms of medical and nursing educational curricula (Andrade, 1978) nor in the public health policies (Atun et al., 2015) among them. This has been evidenced by findings among Latin American physicians and nurses regarding professionalism, measured first from the scope of empathy (Alcorta-Garza et al., 2016), and most recently from the assessment of attitudes toward inter-professional collaborative work and lifelong learning (San-Martín et al., 2017a). With regard to inter-professional relationships, and specifically to the role that professionals play, two studies in Mexican healthcare institutions were the first to report the prevalence of a “hierarchical” model in physician-nurse work relationships in Latin America (Hojat et al., 2001, 2003). Fifteen years later, two international studies, one carried out among healthcare professionals who worked at public institutions in four Latin American countries (San-Martín et al., 2017a), and the other regarding Latin American medical students enrolled in training programs at Spanish teaching hospitals (San-Martín et al., 2017b), were published. Both studies not only confirmed the pervasiveness of a hierarchical model in the physician-nurse work relationships of new generations of Latin American healthcare professionals, but also remarked on the important role that some cultural stereotypes, mainly associated with professional roles, play in the development of three critical aspects of professionalism: empathy, inter-professional collaborative work, and lifelong learning.

Based on these findings, this study was designed in a Mexican university with two main objectives. The first was to confirm whether differences related to professional roles, previously reported in Mexico between physicians and nurses, are already evident in first-year medical and nursing students at two time-points, during the first month of their studies and upon completion of the first academic semester (4 months). According to this first objective, two research hypotheses were established. The first hypothesis was that first-year Mexican students attending medical and nursing schools develop different attitudes (that can be psychometrically measured) very early on, regarding the dynamics of physician-nurse collaborative work, due to the influence that culture and social stereotypes play on professional roles. The second hypothesis was that the differences observed in the development of attitudes toward physician-nurse collaborative work among students are not related to sex differences.

The second objective of this study was to prove the efficacy of a training program, based on coaching methodology, to improve attitudes toward physician-nurse collaborative work in first-year students enrolled in Mexican medical and nursing schools. A closer look at this second objective led to the creation of two more research hypotheses. The third one was that first-year students who attended a training program on inter-professional collaborative work improved their inter-professional collaboration abilities in physician-nurse teamwork. The fourth hypothesis was that a greater development of these inter-professional work abilities had a positive effect on the development of empathy and lifelong learning, given that these three areas comprise the core elements of professionalism.

## Materials and Methods

### Participants

Medical and nursing students from of the Autonomous University of Coahuila, in Piedras Negras, Mexico participated in the study. Two first-year undergraduate student cohorts, one of medical students from the School of Medicine and another of nursing students from the School of Health Sciences, were invited to participate in the study at the beginning of their first academic semester. Of these, 183 students (77 male, 106 female), representing 59% of both cohorts, agreed to participate.

### Design and Procedure

**FIGURE 1:**
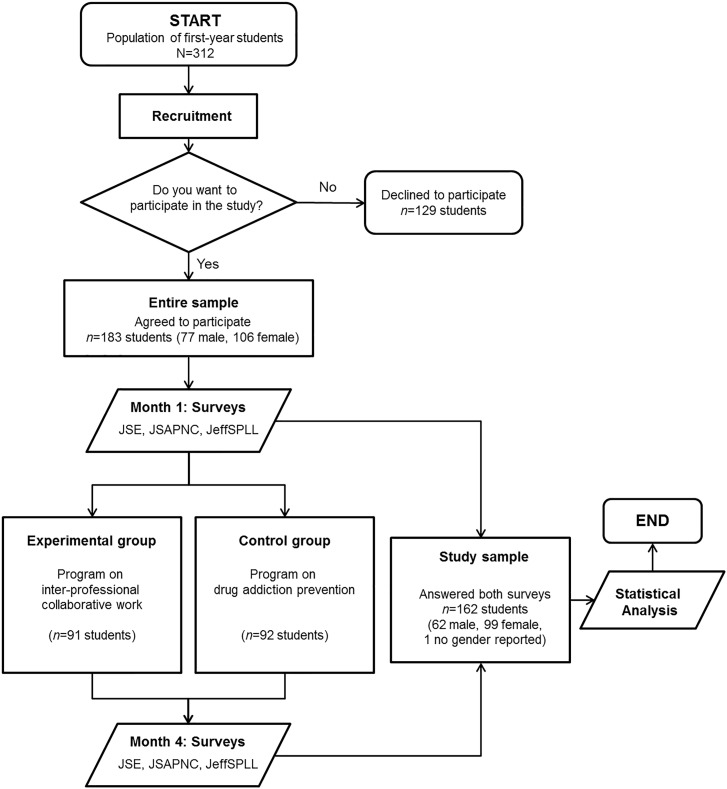
Schematic overview of the workflow diagram followed in this study. JSAPNC, Jefferson Scale of Attitudes toward Physician-Nurse Collaboration; JSE, Jefferson Scale of Empathy (JSE-S, Medical Student version and JSE-HPS, Health Professions Student version); JeffSPLL, Jefferson Scale of Lifelong Learning (JeffSPLL-MS, Medical Student version and JeffSPLL-HPS, Health Professions Student version).

In the first week of class, two cohorts of first-year students (one from the medical school and the other from the nursing school, both from the same university) attended a workshop led by an external researcher. In the workshop, students were informed that this study was performed for the purpose of measuring the development of some competencies associated with professionalism in healthcare workers. Participants were randomly divided into two groups, maintaining a similar distribution according to their degree coursework and sex, in accordance with an experimental design previously developed. One group (experimental group) received a 4-month training program on inter-professional collaborative work abilities, whereas the other one (control group) received a 4-month training program on drug addiction prevention. A multiple-choice questionnaire including specific psychometric measurements (see below) was administered before and after the training programs. The questionnaires were on paper and were returned in sealed envelopes. The students’ participation was anonymous, and this anonymity was maintained through the use of pseudonyms. There was no potential harm to participants. Finally, those participants who completed both groups of questionnaires were included in the statistical analysis. A summary of the study design is shown in Figure 1.

An independent ethical committee for clinical research (Comité Ético de Investigación Clínica de La Rioja) approved the study design (Ref. CEICLAR PI 199) in Spain. The study was carried out in accordance with recommendations and authorization of the participating institution’s administration (Autonomous University of Coahuila, Piedras Negras). In accordance with the Declaration of Helsinki, all students who agreed to participate in this study brought a written informed consent.

### Main Measures

The Jefferson Scale of Attitudes toward Physician-Nurse Collaboration (JSAPNC) was used. The JSAPNC was developed in response to the need for a valid instrument to measure an important aspect of professionalism, namely teamwork and inter-professional collaboration between physicians and nurses. According to the authors of the JSAPNC (Hojat et al., 1999), physician-nurse collaboration is defined as an ability of nurses and physicians to work together cooperatively, sharing responsibilities for solving problems and making decisions to formulate and carry out plans for patient care. The JSAPNC is composed of 15 Likert scale items that are scored from 1 (strongly disagree) to 4 (strongly agree). This instrument has demonstrated a high reliability and validity among medical and nursing students in different cultural contexts (Ward et al., 2008; Hojat et al., 2015; Zakerimoghadam et al., 2015).

Two versions of the Jefferson Scale of Empathy (JSE) were used to measure students’ orientation toward empathetic relationships with patients (Hojat, 2016): the medical student version (JSE-S), for medical students and the healthcare student version (JSE-HPS) for nursing students. The JSE has 20 Likert scale-type items worth seven points, ranging from 1 (strongly disagree) to 7 (strongly agree). The difference between the two versions of the JSE used is in the rewording of those items where the terms “medicine” or “physician” appeared, in order to make it more applicable to students from other healthcare areas. A sample item of the JSE-S is: “Physicians should try to stand in their patient’s shoes when providing care to them,” while the same item in the JSE-HPS is reworded as follows: “Health care providers should try to stand in their patients’ shoes when providing care to them.” A higher score means that the student has a greater orientation or behavioral tendency toward empathic engagement in patient care (Hojat, 2016).

Two versions of the Jefferson Scale of Physician Lifelong Learning (JeffSPLL) were used to measure attitudes toward lifelong learning: the medical student version (JeffSPLL-MS), for medical students (Wetzel et al., 2010); and the healthcare profession student version (JeffSPLL-HPS) for nursing students (Novak et al., 2014). The JeffSPLL was originally designed to measure the development of skills related to information gathering, the use of learning opportunities, and self-motivation (Hojat et al., 2009). The JeffSPLL has 14 Likert scale-type items that are scored from 1 (strongly disagree) to 4 (strongly agree). Similarly to the two versions of the empathy scale, the differences between the two versions of the JeffSPLL are in the rewording of those items where the term “medicine” is used, in order to make it more applicable to students from other healthcare areas. The JeffSPLL tool has demonstrated a high reliability and validity in both student groups in different cultural contexts (Muliira et al., 2012; San-Martín et al., 2016).

Information regarding degree coursework, sex, and age were collected through a complementary form.

### Training Programs

Based on coaching methodology, two extra-curricular training programs were specially developed for this study: (i) for the experimental group, a program on inter-professional collaborative work; and (ii) for the control group, a program on drug addiction prevention. Both programs included similar methodologies based on theoretical classes and practical sessions, the same number of lessons (36 lecture hours) distributed over 18 weekly workshops of 2 h each, and were offered in parallel during the first academic semester (4 months). A detailed description of the contents of each program is reported in Appendix 1.

### Statistical Analysis

The reliability of the instruments used was measured by Cronbach’s alpha coefficient. This assessment was performed twice: before and after the training programs were given.

Once the normality of the inter-professional collaborative work scores was studied, a variance analysis (Three-way ANOVA) based on the following variables was performed, both prior to and at the end of the study: study group, sex, degree coursework, and the interaction between sex and degree coursework. Furthermore, a comparative analysis using Wilcoxon tests, by study groups (experimental vs. control) and by degree coursework (medicine vs. nursing), before and after the study was performed. In order to determine possible associations among inter-professional collaborative work abilities, empathy, and lifelong learning, a correlation analysis using Spearman’s coefficient (*ρ*) among the above-mentioned variables was also performed at the beginning and at the end of the study. Finally, a multiple regression analysis for “study group by degree coursework” variables was performed in order to determine whether the development of inter-professional collaborative work abilities at the end of the course has a different effect on the other two measured components of professionalism: empathy and lifelong learning.

Data processing was carried out with R software (R Core Team, 2017), version 3.4.1 for Windows, and included the use of *nortest* (Gross and Ligges, 2015) and *multilevel* (Bliese, 2016) statistical packages.

## Results

### Participants

From the entire sample of 183 students, 162 students (62 males, 99 females, and one gender not stated) responded to both questionnaires. This sample was used for the statistical analysis and represented 52% of the entire population of first-year students enrolled in medical and nursing programs offered by the School of Medicine and by the School of Healthcare Sciences of the Autonomous University of Coahuila, in Piedras Negras, Mexico.

According to their degree coursework, 84 participants were first-year medical students (45 males, 38 females, and two genders not declared), and 89 were first-year nursing students (26 males and 63 females). The mean age of the entire sample was 19 years, ranging from 18 to 35 years (*SD* = 2).

### Reliability and Descriptive Statistics

The range observed for Cronbach’s alpha coefficients obtained for the five instruments used in this study was between 0.71 and 0.80. The test-retest reliability over a 4-month period yielded values ranging between 0.60 and 0.83. The score distribution summary, and reliability for all instruments used are reported in Table 1.

**Table 1:** Descriptive analysis, reliability, and reliability of the Jefferson Scales of physician-nurse collaboration, empathy, and lifelong learning.

	Start			End (4-month)		
	*n*	M (*SD*)	Reliability	*n*	M (*SD*)	Reliability
JSAPNC	159	48 (6)	0.72	154	48 (7)	0.82
JSE-S	76	104 (12)	0.72	75	100 (14)	0.75
JSE-HPS	77	96 (13)	0.77	77	97 (11)	0.60
JeffSPLL-MS	78	46 (4)	0.71	76	46 (6)	0.83
JeffSPLL-HPS	76	44 (5)	0.80	82	45 (5)	0.68


*JSAPNC, Jefferson Scale of Attitudes toward Physician-Nurse Collaboration; JSE-S, Jefferson Scale of Empathy- Medical Student version; JSE-HPS, Jefferson Scale of Empathy-Health Professions Student version; JeffSPLL-MS, Jefferson Scale of Physician Lifelong Learning-Medical Student version; JeffSPLL-HPS, Jefferson Scale of Physician Lifelong Learning-Health Professions Student version; *n*, sample size; M, mean; *SD*, standard deviation.*

### Measurement of Attitudes Toward Physician-Nurse Collaboration in First-Year Medical and Nursing Students

The first hypothesis which looked at the influential role that culture plays in students’ attitudes toward inter-professional work showed no differences in JSAPNC of newly admitted students when compared by degree coursework (*p* = 0.06). However, an inverse association between JSAPNC and age was confirmed (*ρ* = -0.17; *p* = 0.03), but only in newly admitted students. The second time that the JSAPNC was measured, just after the study was finished, this association disappeared (*ρ* = +0.003; *p* = 0.97). On the other hand, the second measurement of the JSAPNC showed new differences according to study group (*p* = 0.0005), and degree coursework (*p* = 0.00004), as shown in Table 2.

**Table 2:** Descriptive statistics and analysis of variance (Three-way ANOVA) of the scores of the inter-professional collaborative work by study group, gender, and degree coursework (*n* = 162).

	Before					After			
	*n*	M (*SD*)	*F*(1,154)	η^2^	*p*	M (*SD*)	*F*(1,149)	η^2^	*p*
Study group			2.08	0.01	0.14		11.69	0.07	<0.001
Control	82	47 (5)				46 (7)			
Experimental	80	48 (6)				50 (5)			
Sex			3.54	0.02	0.16		0.04	0.00	0.35
Male	62	48 (6)				47 (7)			
Female	99	47 (5)				48 (6)			
Degree coursework			3.66	0.02	0.06		17.95	0.11	<0.001
Medicine	78	47 (6)				46 (7)			
Nursing	83	48 (5)				50 (6)			


*n, sample size; M, mean; SD, standard deviation; F, F-value; η^2^, Eta-squared; p, p-value.*

Regarding the second research hypothesis, no differences were found when the JSAPNC score was compared by gender, both at beginning (*p* = 0.16) and at the end (*p* = 0.35) of the study. A similar situation was also observed for the interaction between sexes and degree coursework, both at beginning (*p* = 0.805) and at the end (*p* = 0.841).

### Enhancement of Attitudes Toward Physician-Nurse Collaboration in First-Year Medical and Nursing Students

The third research hypothesis related to the positive effect of a training program on inter-professional collaborative work abilities. A three-way ANOVA, at the beginning and at the end, revealed that students from the experimental group achieved higher scores on the JSAPNC compared to those who participated in the control group, and this difference was statistically significant (*p* = 0.0005), as shown in Table 2. According to degree coursework, the comparative analysis by study groups showed that JSAPNC scores of nursing students from experimental group increased over time (*p* = 0.02), while JSANPC scores of medical students of this group remained stable (*p* = 0.57). However, in the control group, JSPANC scores of medical students showed a significant deterioration over the time (*p* = 0.006), while nursing students of this group remained stable (*p* = 0.16). The summary of these findings is reported in Table 3.

**Table 3:** Summary result of Wilcoxon test of JSAPNC scores by study groups (experimental vs. control) before and after the study was performed.

Study group	*n*	Before		After		*p*
		M	*SD*	M	*SD*	
**Control group**						
Medicine	37	47	6	44	7	0.006
Nursing	38	47	5	49	7	0.16
**Experimental group**						
Medicine	34	47	7	48	5	0.57
Nursing	42	50	4	51	5	0.02


*JSAPNC, Jefferson Scale of Attitudes toward Physician-Nurse Collaboration; *n*, sample size; M, mean; *SD*, standard deviation; *p*, *p-*value.*

**FIGURE 2:**
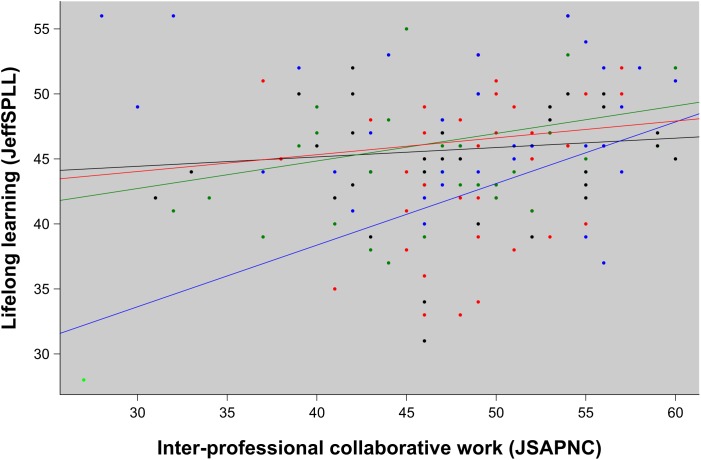
Regression analysis of lifelong learning abilities (measured by the JeffSPLL), according to inter-professional collaborative work (measured by the JSAPNC). For “study group by degree coursework” groups: in red, medical students in the experimental group; in blue, nursing students of the experimental group; in green, medical students of the control group; and in black, nursing students of the control group (*p =* 0.028).

The fourth research hypothesis related to the role that inter-professional abilities play in the development of the other two measured elements of professionalism. Correlation analysis of the entire sample confirmed a positive relationship between empathy and inter-professional collaboration, both at the beginning (*ρ* = +0.27; *p* = 0.0008) and at the end (*ρ* = +0.41; *p* < 0.0001) of the study. Although no association was observed between lifelong learning and teamwork in newly admitted students (*ρ* = +0.11; *p* = 0.17), a significant association between these two elements appeared at the end of the study (*ρ* = +0.19; *p* = 0.02). At this point, statistical differences were also observed between inter-professional collaborative work and lifelong learning according to “study group by degree coursework” (*p* = 0.03). Those differences are shown in Figure 2.

## Discussion

The scales showed adequate psychometric properties with Cronbach’s alpha coefficients higher than the international recommendation of 0.70 set by the American Educational Research Association. Results observed in the present study support the use of the Spanish versions of the above-mentioned scales as consistent measures of empathy, teamwork, and lifelong learning for medical and nursing students. These findings are also similar to those previously reported for empathy in medical and nursing students (Hojat, 2016), and slightly lower than those reported for teamwork and lifelong learning (Hojat et al., 1999; Wetzel et al., 2010; Novak et al., 2014).

Regarding the first objective, teamwork, and cultural influence, the inverse association found between teamwork and age in newly admitted students suggests that students who start their degree coursework at a later age have a higher risk of presenting certain prejudgments related to professional roles and inter-professional work than in those who begin at a younger age. The most plausible explanation for these findings is the negative effect of dominant cultural stereotypes related to professional roles, a finding that is in accordance with research evidence reported in previous studies of Mexican (Hojat et al., 2001, 2003) and other Latin American healthcare professionals (San-Martín et al., 2017a,b). The findings of this study regarding the increasing difference in the development of this ability between medical and nursing students over time, or the deterioration in the development of this ability in the control group provide new evidence supporting the negative influence of the “hierarchical” dominant model in physician-nurse work relationships. The findings reported in this study clearly confirm that differences reported for inter-professional collaborative work among students are due to their degree coursework and not to their gender differences. This is also consistent with other studies where those differences were directly associated with professional roles (San-Martín et al., 2017a).

On the other hand, the study demonstrates that, similar to previously reported findings for empathy (Cunico et al., 2012; Hojat, 2016), collaborative inter-professional work can be enhanced and sustained in medical and nursing students by targeted educational programs. The significant increase in JSAPNC scores by the nursing students in the experimental group who were attending the training program, or the stability in the JSANPC scores of the medical students who were participating in the experimental group, evidence an antagonist tendency in comparison with those observed in medical and nursing students who were enrolled in the control group. These findings resonate with those reported in a recent study where there was a significant overlap between empathy, teamwork, and integrative patient care. The authors concluded that an improvement in inter-professional work skills is not only possible but can also positively influence the improvement of other components of medical professionalism, such as empathy (Hojat et al., 2015). In accordance with this last issue, the findings of this study confirm the positive influence that improvement of teamwork has on the other two components of professionalism. Along the same lines, a strong relationship among empathy, teamwork, and lifelong learning are experimentally demonstrated. These findings coincide with others reported in previous studies with medical students (San-Martín et al., 2016) and healthcare professionals (San-Martín et al., 2017a,b; Soler-González et al., 2017). In all these studies, new research evidence supports a multi-score measurement of medical professionalism, initially proposed by Veloski and Hojat (2006) in clinical settings with a cross-cultural scope. This study supplies new evidence in an undergraduate population. Additionally, this study provides new evidence supporting the importance of improving inter-professional skills in the development of attitudes toward lifelong learning in nursing students. This issue is especially remarkable given that the development of lifelong learning skills as a professional competence is an important gap in nurses who are working under the influence of dominant “hierarchical” work models, as has been reported in recent studies in healthcare institutions located in developing countries in Africa (Muliira et al., 2012) and Latin America (San-Martín et al., 2017a).

## Author Contributions

LV was in charge of the study’s overall design. GT-G oversaw development of the training programs and carried out the study in the participating institutions in Mexico. LV and MS-M statistically processed the data. LV and RD-B prepared the draft manuscript. All authors contributed to the present work, participated in the interpretation and processing of results, and reviewed and approved the final manuscript.

## Conflict of Interest Statement

The authors declare that the research was conducted in the absence of any commercial or financial relationships that could be construed as a potential conflict of interest.
