# Supplementary material for: Improvement of Inter-Professional Collaborative Work Abilities in Mexican Medical and Nursing Students: A Longitudinal Study
Source: Front Psychol. 2019 Jan 15;10:5. doi: 10.3389/fpsyg.2019.00005 (PMC6340986; doi:10.3389/fpsyg.2019.00005)
Supplement: Supplementary file 1 [file Data_Sheet_1.docx]

**Appendix 1**

**Training program on inter-professional collaborative work**

Contents:

Session 1: Learning emotional intelligence and the proper use of emotions

Session 2: Assertive communication

Session 3: Anger, fear, anxiety, relaxation, sadness

Session 4: Congruence, empathy, self-esteem and respect

Session 5: Ladder of Inference

Session 6: Positive side of Motivation

Session 7: Shared Vision

Session 8: The power of inter-professional collaborative work

Session 9: Learning and designing in collaboration

Session 10: Systemic thinking

Session 11: Concept and orientation in the study of personality

Session 12: Sense of belonging

Session 13: Highly effective collaborators

Session 14: Types and keys of leadership

Session 15: Agents of change

Session 16: Organizational philosophy

Session 17: Self-realization at the work place

Session 18: Being, knowledge, and know-how

**Training program on drug addiction prevention**

Contents:

Session 1: Introduction to the problem of addiction

Session 2: Problematic Use

Session 3: Pharmacological basis of addiction

Session 4: Consumption statistics

Session 5: Toxic and non-toxic, legal and illegal addictions

Session 6: Basis for consumption prevention

Session 7: Prevention strategy

Session 8: Diagnostic levels

Session 9: Diagnosis and therapeutic decisions

Session 10: Co-dependence

Session 11: System of the addict patient and the patient’s family

Session 12: Family recovery program

Session 13: Acceptance of the problem

Session 14: Abstinence, help and tools

Session 15: Recovery

Session 16: Emotional relapse and mental behavior

Session 17: Relapse in the family

Session 18: New technological addictions
